# Supplementary material for: Development Trajectories of Fatigue, Quality of Life, and the Ability to Work among Colorectal Cancer Patients in the First Year after Rehabilitation—First Results of the MIRANDA Study
Source: Cancers (Basel). 2023 Jun 13;15(12):3168. doi: 10.3390/cancers15123168 (PMC10296005; doi:10.3390/cancers15123168)
Supplement: Supplementary file 1 [file cancers-15-03168-s001.zip › cancers-2445746-supplementary.pdf]

Supplemental Material

Development trajectories of fatigue, quality of life, and the ability to work among colorectal cancer patients in the first year after rehabilitation – First results of the MIRANDA study

Table of contents

Suppl. Table S1. Cross-tabulation of the categories of the FACIT-F-FWB-ability to work (AW) item and the Work Ability Index (WAI) 2

Suppl. Table S2. Comparison of important baseline characteristics of participants of all follow-ups and individuals who dropped out of the study by non-response to at least one follow-up questionnaire........ 3

Suppl. Figure S1. Trajectories of FACIT-F-FS (A), EORTC-QLQ-C30-GHS/QoL (B), and FACIT-F-FWB-AW (C) from baseline to 12-month follow-up by sex........ 4

Suppl. Figure S2. Trajectories of FACIT-F-FS (A), EORTC-QLQ-C30-GHS/QoL (B), and FACIT-F-FWB-AW (C) from baseline to 12-month follow-up by age 5

Suppl. Figure S3. Trajectories of FACIT-F-FS (A), EORTC-QLQ-C30-GHS/QoL (B), and FACIT-F-FWB-AW (C) from baseline to 12-month follow-up by stage 6

Suppl. Figure S4. Trajectories of FACIT-F-FS (A), EORTC-QLQ-C30-GHS/QoL (B), and FACIT-F-FWB-AW (C) from baseline to 12-month follow-up by participation in the VICTORIA study........ 7

Suppl. Figure S5. Trajectories of FACIT-F-FS (A), EORTC-QLQ-C30-GHS/QoL (B), and FACIT-F-FWB-AW (C) from baseline to 12-month follow-up by time since surgery 8

Suppl. Table S1. Cross-tabulation of the categories of the FACIT-F-FWB-ability to work (AW) item and the Work Ability Index (WAI)

FACIT-F-FWB-AW	WAI index			Total n (%)
	1	2	3-4	
1-3	3 (6.1%)	7 (14.3%)	2 (4.1%)	12 (24.5%)
4	1 (2%)	13 (26.5%)	3 (6.1%)	17 (34.7%)
5	0 (0%)	11 (22.5%)	9 (18.3%)	20 (40.8%)
Total n (%)	4 (8.2%)	31 (63.3%)	14 (28.6%)	49 (100%)[a]

Abbreviations: FACIT-F-FWB-AW, Functional Assessment of Chronic Illness Therapy – Fatigue – Functional Well-Being – Ability to Work item; WAI, Work Ability Index

[a] Only study participants who worked at the 9-month follow-up could be included in this analysis (N=49) because the WAI can only be answered by individuals who are currently at work.

Suppl. Table S2. Comparison of important baseline characteristics of participants of all follow-ups and individuals who dropped out of the study by non-response to at least one follow-up questionnaire

	Response to all follow-up questionnaires (N=114)	Non-response to at least one follow-up questionnaire (N=33)	P value [a]
	Proportion (%)	Proportion (%)	
**Age (years)**			
< 65	71 (62.3)	19 (57.6)	0.625
≥ 65	43 (37.7)	14 (42.4)	
**Sex**			
Female	41 (35.9)	14 (47.4)	0.500
Male	73 (64.0)	19 (57.6)	
**Cancer stage**			
I	37 (32.5)	12 (36.4)	0.737
II	37 (32.5)	8 (24.2)	
III	27 (23.7)	10 (30.3)	
Unknown	13 (11.4)	2 (6.1)	
**Participation in VICTORIA study**			
Yes	55 (48.3)	18 (45.5)	0.524
No	59 (51.7)	15 (54.5)	

[a] Chi square test

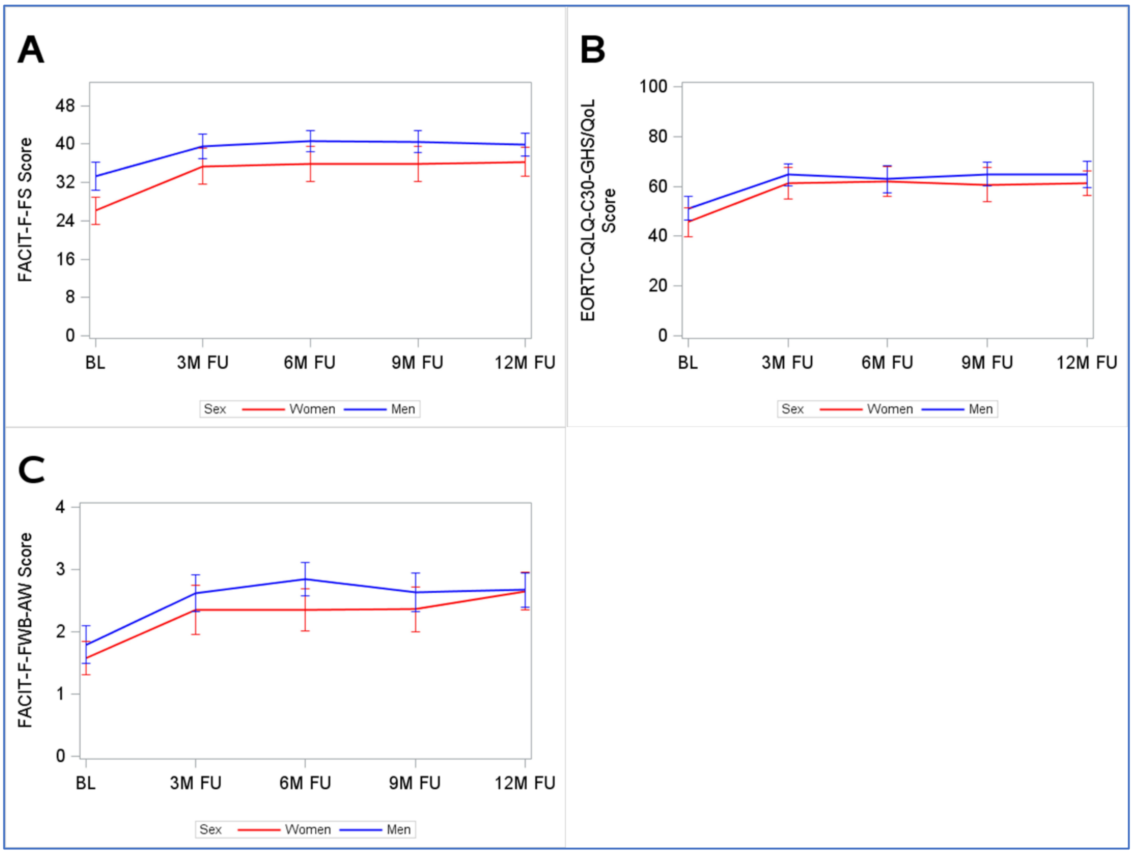

Suppl. Figure S1. Trajectories of FACIT-F-FS (A), EORTC-QLQ-C30-GHS/QoL (B), and FACIT-F-FWB-AW (C) from baseline to 12-month follow-up by sex

Abbreviations: BL, baseline; 3M FU, 3-month follow-up; 6M FU, 6-month follow-up; 9M FU, 9-month follow-up; 12M FU, 12-month follow-up

Note: The figure displays means with 95% confidence intervals.

Only study participants with complete follow-up data were included in this analysis (n=114).

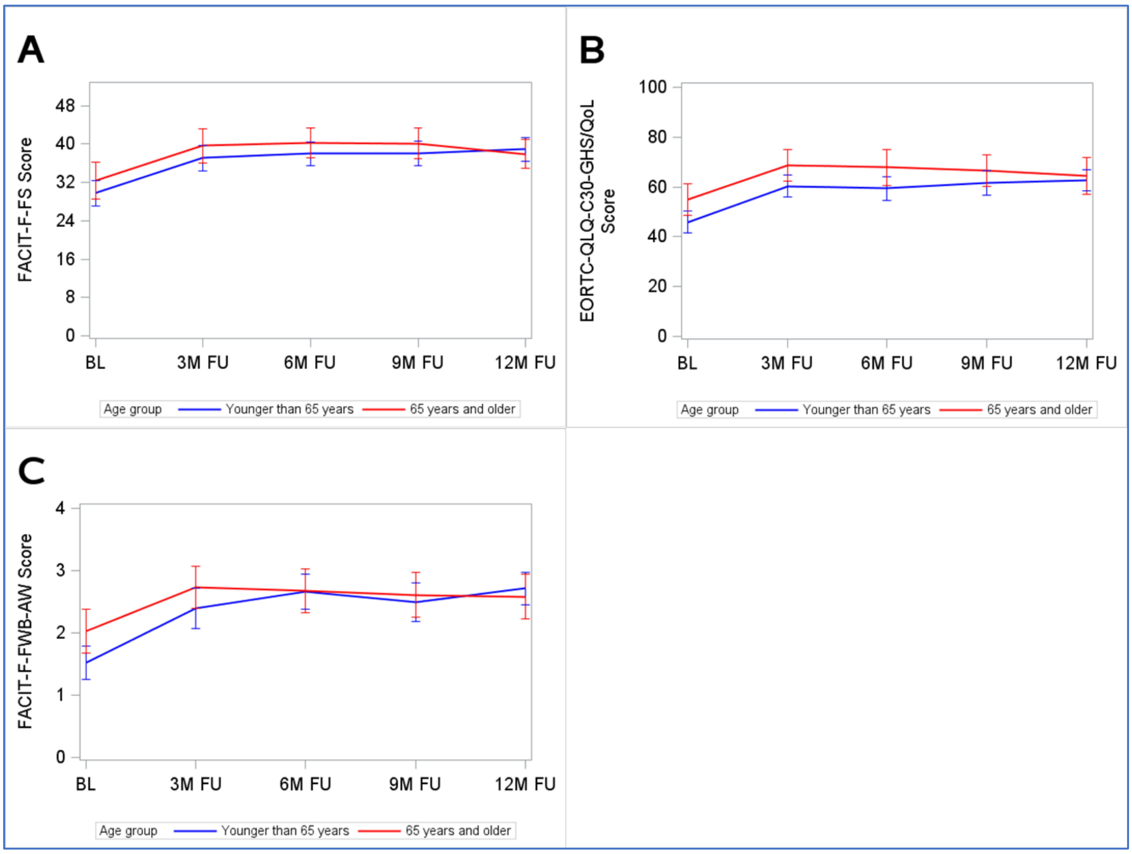

Suppl. Figure S2. Trajectories of FACIT-F-FS (A), EORTC-QLQ-C30-GHS/QoL (B), and FACIT-F-FWB-AW (C) from baseline to 12-month follow-up by age

Abbreviations: BL, baseline; 3M FU, 3-month follow-up; 6M FU, 6-month follow-up; 9M FU, 9-month follow-up; 12M FU, 12-month follow-up

Note: The figure displays means with 95% confidence intervals.

Only study participants with complete follow-up data were included in this analysis (n=114).

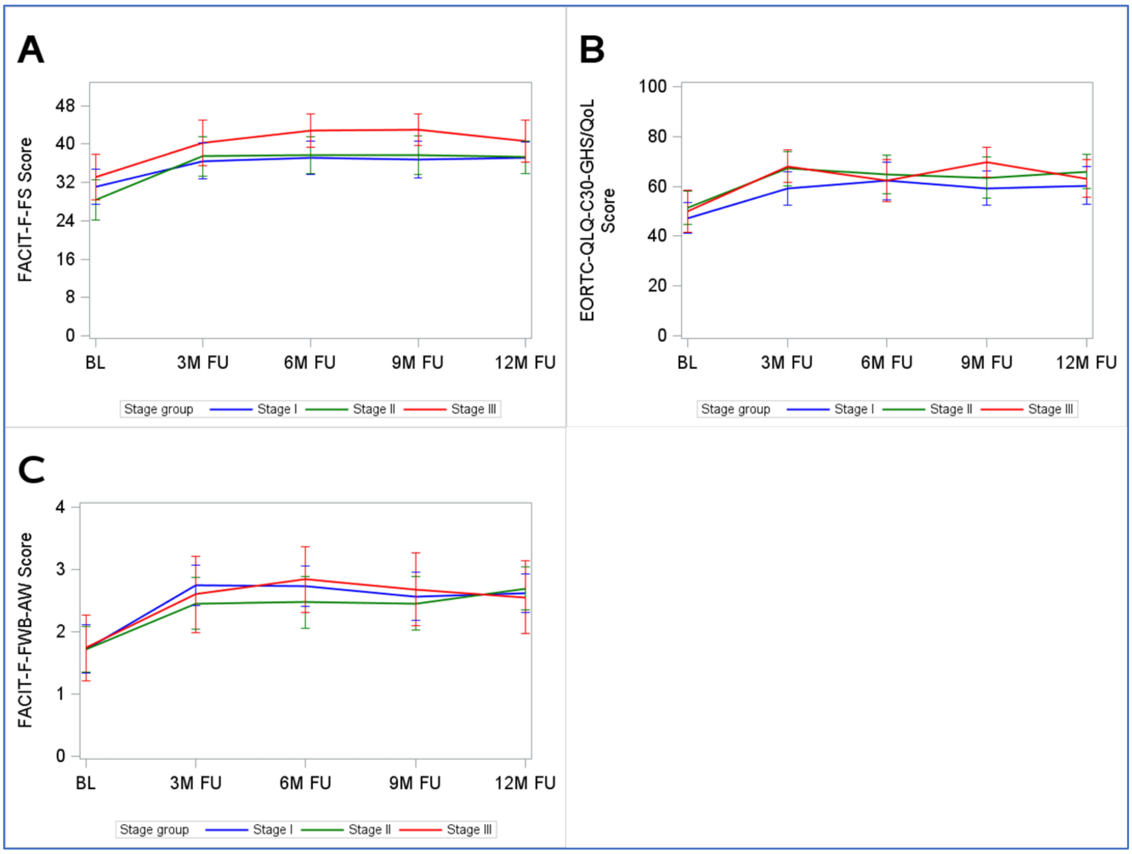

Suppl. Figure S3. Trajectories of FACIT-F-FS (A), EORTC-QLQ-C30-GHS/QoL (B), and FACIT-F-FWB-AW (C) from baseline to 12-month follow-up by stage

Abbreviations: BL, baseline; 3M FU, 3-month follow-up; 6M FU, 6-month follow-up; 9M FU, 9-month follow-up; 12M FU, 12-month follow-up

Note: The figure displays means with 95% confidence intervals.

Only study participants with complete follow-up data were included in this analysis (n=114).

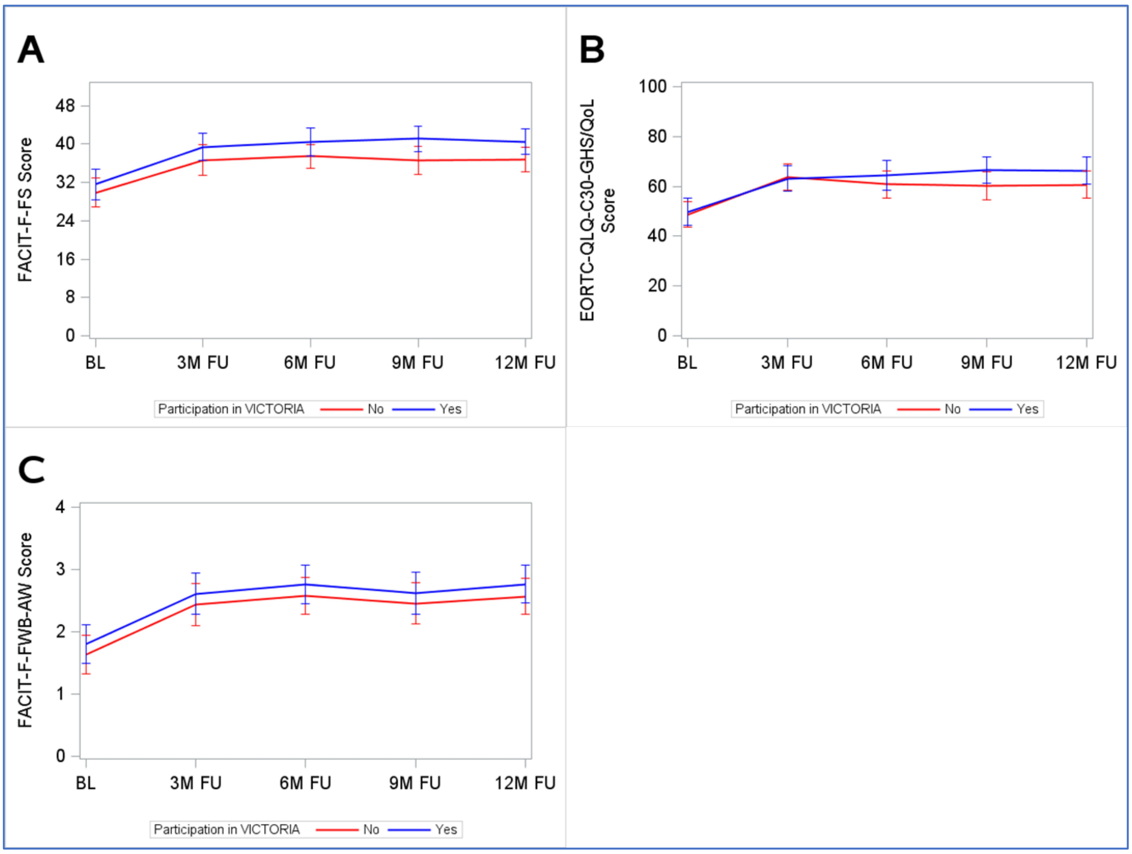

Suppl. Figure S4. Trajectories of FACIT-F-FS (A), EORTC-QLQ-C30-GHS/QoL (B), and FACIT-F-FWB-AW (C) from baseline to 12-month follow-up by participation in the VICTORIA study

Abbreviations: BL, baseline; 3M FU, 3-month follow-up; 6M FU, 6-month follow-up; 9M FU, 9-month follow-up; 12M FU, 12-month follow-up

Note: The figure displays means with 95% confidence intervals.

Only study participants with complete follow-up data were included in this analysis (n=114).

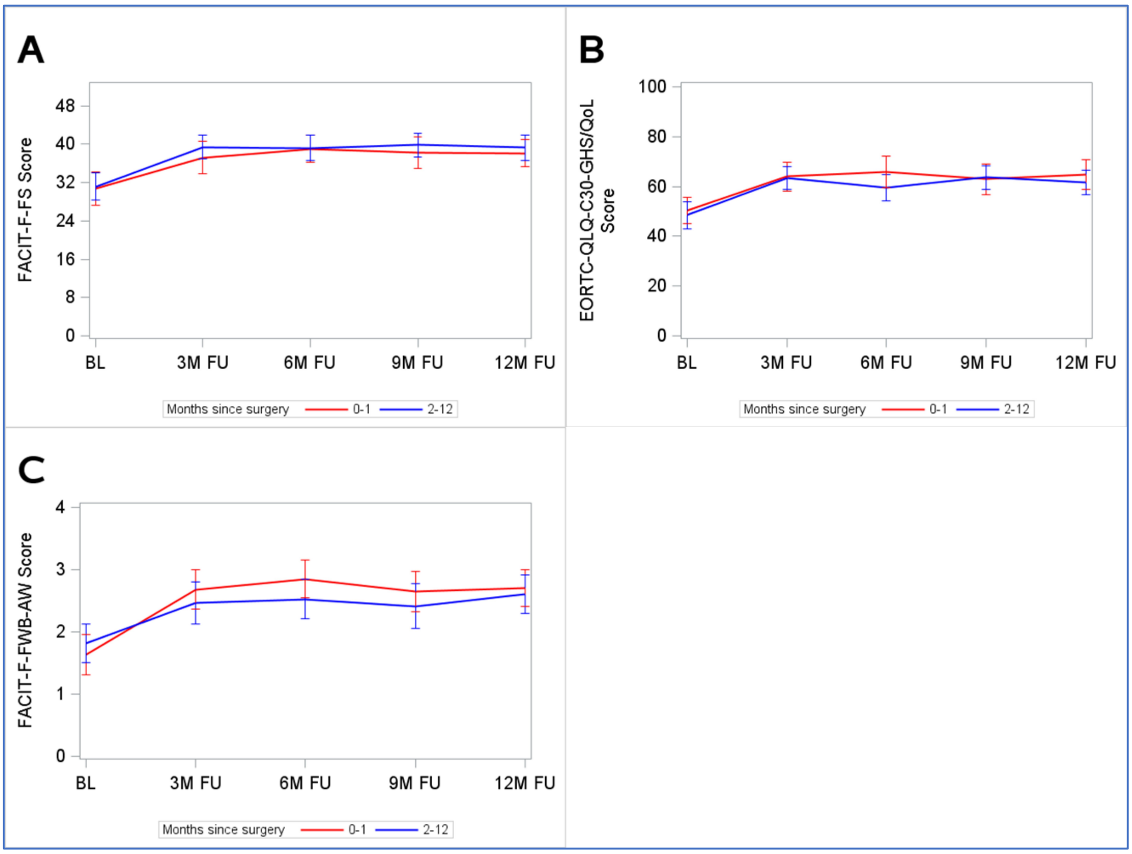

Suppl. Figure S5. Trajectories of FACIT-F-FS (A), EORTC-QLQ-C30-GHS/QoL (B), and FACIT-F-FWB-AW (C) from baseline to 12-month follow-up by time since surgery

Abbreviations: BL, baseline; 3M FU, 3-month follow-up; 6M FU, 6-month follow-up; 9M FU, 9-month follow-up; 12M FU, 12-month follow-up

Note: The figure displays means with 95% confidence intervals.

Only study participants with complete follow-up data were included in this analysis (n=114).
